# Coordination of olfactory receptor choice with guidance receptor expression and function in olfactory sensory neurons

**DOI:** 10.1371/journal.pgen.1007164

**Published:** 2018-01-31

**Authors:** Puneet Dang, Stephen A. Fisher, Derek J. Stefanik, Junhyong Kim, Jonathan A. Raper

**Affiliations:** 1 Department of Neuroscience, Perelman School of Medicine, University of Pennsylvania Philadelphia, PA, United States of America; 2 Department of Biology, School of Arts and Sciences, University of Pennsylvania, Philadelphia, PA, United States of America; Fred Hutchinson Cancer Research Center, UNITED STATES

## Abstract

Olfactory sensory neurons choose to express a single odorant receptor (OR) from a large gene repertoire and extend axons to reproducible, OR-specific locations within the olfactory bulb. This developmental process produces a topographically organized map of odorant experience in the brain. The axon guidance mechanisms that generate this pattern of connectivity, as well as those that coordinate OR choice and axonal guidance receptor expression, are incompletely understood. We applied the powerful approach of single-cell RNA-seq on newly born olfactory sensory neurons (OSNs) in young zebrafish larvae to address these issues. Expression profiles were generated for 56 individual Olfactory Marker Protein (OMP) positive sensory neurons by single-cell (SC) RNA-seq. We show that just as in mouse OSNs, mature zebrafish OSNs typically express a single predominant OR transcript. Our previous work suggests that OSN targeting is related to the OR clade from which a sensory neuron chooses to express its odorant receptor. We categorized each of the mature cells based on the clade of their predominantly expressed OR. Transcripts expressed at higher levels in each of three clade-related categories were identified using Penalized Linear Discriminant Analysis (PLDA). A genome-wide approach was used to identify membrane-associated proteins that are most likely to have guidance-related activity. We found that OSNs that choose to express an OR from a particular clade also express specific subsets of potential axon guidance genes and transcription factors. We validated our identification of candidate axon guidance genes for one clade of OSNs using bulk RNA-seq from a subset of transgene-labeled neurons that project to a single protoglomerulus. The differential expression patterns of selected candidate guidance genes were confirmed using fluorescent *in situ* hybridization. Most importantly, we observed axonal mistargeting in knockouts of three candidate axonal guidance genes identified in this analysis: *nrp1a*, *nrp1b*, and *robo2*. In each case, targeting errors were detected in the subset of axons that normally express these transcripts at high levels, and not in the axons that express them at low levels. Our findings demonstrate that specific, functional, axonal guidance related genes are expressed in subsets of OSNs that that can be categorized by their patterns of OR expression.

## Author summary

Assembling the brain circuitry responsible for our sense of olfaction is a formidable task. The sensory neurons that detect odorants in the nasal epithelium, the olfactory sensory neurons, choose to express a single odorant receptor from a large gene repertoire. Sensory neurons that choose a particular odorant receptor are intermixed with other neurons that have chosen different receptors. Remarkably, all the sensory neurons that choose a particular odorant receptor project axons to the same target region in the brain, while those choosing to express other odorant receptors project axons to different target regions. Presumably, this specificity in axon targeting is related to the differential expression of guidance related genes in subsets of sensory neurons. Is there a systematic method for their identification? Here we use single-cell RNAseq to characterize the transcriptional profiles of individual olfactory sensory neurons. We identify candidate guidance related genes whose expression is correlated to the simultaneous expression of particular odorant receptors. We show in loss of function studies that some of these guidance related genes are required for the guidance of specific subsets of olfactory sensory neurons. This general approach may open the door to the systematic identification of genes that guide axons to their specific targets during the assembly of developing neural circuits.

## Introduction

The development of the nervous system requires that cell fate specification be coordinated with axonal pathfinding, dendrite formation, and synapse formation. Since specific neuronal populations connect with particular regions in the brain, cellular identity is tightly linked with the expression of distinct axon guidance molecules that regulate target selection. In the developing olfactory system, the cellular identity of olfactory sensory neurons (OSNs) is manifested by the expression of particular odorant receptors (ORs). Each OSN expresses only one OR out of a large repertoire available in the genome [1–4]. In the mature nervous system, OSNs project axons to reproducibly positioned, OR-specific glomeruli in the olfactory bulb [5–7]. Olfactory experience is thus projected onto the brain as a topographic map of glomerular activity [8, 9]. The formation of olfactory circuitry therefore requires a close co-ordination between the expression of particular guidance molecules and particular ORs.

What are some of the molecular mechanisms that lead to the establishment of this pattern of connectivity? In the fly, semaphorin expression in the ventral antenal lobe determines whether entering sensory axons extend into dorsal or ventral regions of the lobe [10]. In the mouse, ventrally expressed Slits induce early arriving, Robo2 expressing OSN axons to target dorsal regions of the olfactory bulb [11, 12]. Further, Sema3F expression in early arriving OSN axons is proposed to restrict later arriving, Nrp2 expressing mouse OSN axons to increasingly more ventral target locations [13]. Segregation of OSN axons into OR specific glomeruli is dependent upon the more granular and mosaic expression of a variety of signaling and adhesion molecules. In the fly, tenurins and tol2-related molecules facilitate matching between specific OSN axons and their appropriate postsynaptic targets [14–15]. In the mouse, the combined activity of adhesion and signaling molecules including kirrels, BIG-2, clustered protocadherins, and ephrinAs are thought to drive OSN axon segregation and glomerulus formation [16–19]. Finally, odorant stimulated activity of OSNs leads to a pruning of mistargeted axons and refinement of glomeruli [20]. In both the fly and the mouse, OR choice and glomerular position are tightly coordinated. In the fly, ORs are dispensable for glomerular targeting and a network of transcription factors are likely to determine both cell identity and guidance receptor expression [21–24]. In contrast, OR expression affects glomerular targeting in the mouse [7, 25]. This has led to the development of a model in which OR activity influences the expression of guidance receptors and adhesion molecules including NRP1 and the kirrels [16, 26].

In spite of this progress in understanding the development of olfactory circuitry, there is still much to learn about how olfactory axons navigate to their appropriate targets, particularly in vertebrate systems. RNA-seq is a valuable approach for the identification of new candidate guidance-related genes. Several studies have examined the transcriptomes of OSNs as revealed by bulk RNA-seq of pooled olfactory sensory neurons [27–32]. Typically the pooled cells were selected using broad markers such as the Olfactory Marker Protein (OMP). This approach has identified axon guidance molecules that are expressed at various stages of neuronal maturation [28]. Single cell RNA-seq studies have revealed that OSNs express multiple OR transcripts early before settling on a single OR [31–34]. However, little attention has been paid to differentially expressed transcripts that could regulate specific axonal targeting of particular OSNs. There is a dearth of markers that label smaller OSN subpopulations within the broader OMP population, making it difficult to identify axon guidance mechanisms through which OSNs achieve differential targeting. It is equally challenging to study the correlation between OR-specific neuronal identity and guidance molecule expression, a fundamental feature of olfactory circuit development.

We used single-cell RNA-seq in the olfactory system of larval zebrafish to address these issues. OSNs are born, differentiate, and extend axons to the bulb starting around 30 hpf [35]. By 72 hpf a crude topographic map is already established in the olfactory bulb with OSN axons targeting 11 discrete, individually identifiable neuropilar regions called protoglomeruli [35]. Our previous work showed that OSNs choosing to express the most related ORs innervate a common protoglomerulus, thus demonstrating an early link between neuronal identity and axon guidance [36]. OSNs choosing to express ORs from OR clades A or B project to the CZ protoglomerulus, while those expressing ORs from OR clade C project to the DZ. What are the molecular mechanisms that enable these nearly identical OMP expressing axons to specifically target either the CZ or the DZ protoglomeruli? We hypothesize that there are distinct, target specific sets of guidance receptors expressed by each category of neuron. Further, since axons that express related ORs converge onto the same protoglomerulus, guidance molecules should be differentially expressed depending upon the OR they express. For these reasons, we set out to identify guidance related molecules that are differentially expressed depending upon the OR an OSN chooses to express.

In this study we show that differentially expressed transcription factors and guidance related molecules can be identified in specific subsets of OSNs expressing related ORs. In this way we identify candidate axonal guidance molecules that have the potential to guide distinct sub-classes of OSNs to specific targets in the bulb. We show that different subsets of axon guidance transcripts show strong correlation with specific subsets of transcription factors, potentially identifying transcriptional pathways that could coordinate neuronal identity with axon guidance. Finally, we provide evidence that three of the candidate guidance genes we identify: *nrp1a*, *nrp1b*, and *robo2;* are functionally required for targeting particular subsets of axons to distinct protoglomeruli. Our findings are consistent with a model of olfactory map formation in which subsets of OSNs express common guidance molecules leading them to target larger protoglomeruluar regions that are subsequently subdivided into smaller OR-specific glomeruli later in development.

## Results

### Cell selection, library preparation, and transcriptome generation

**Fig 1:**
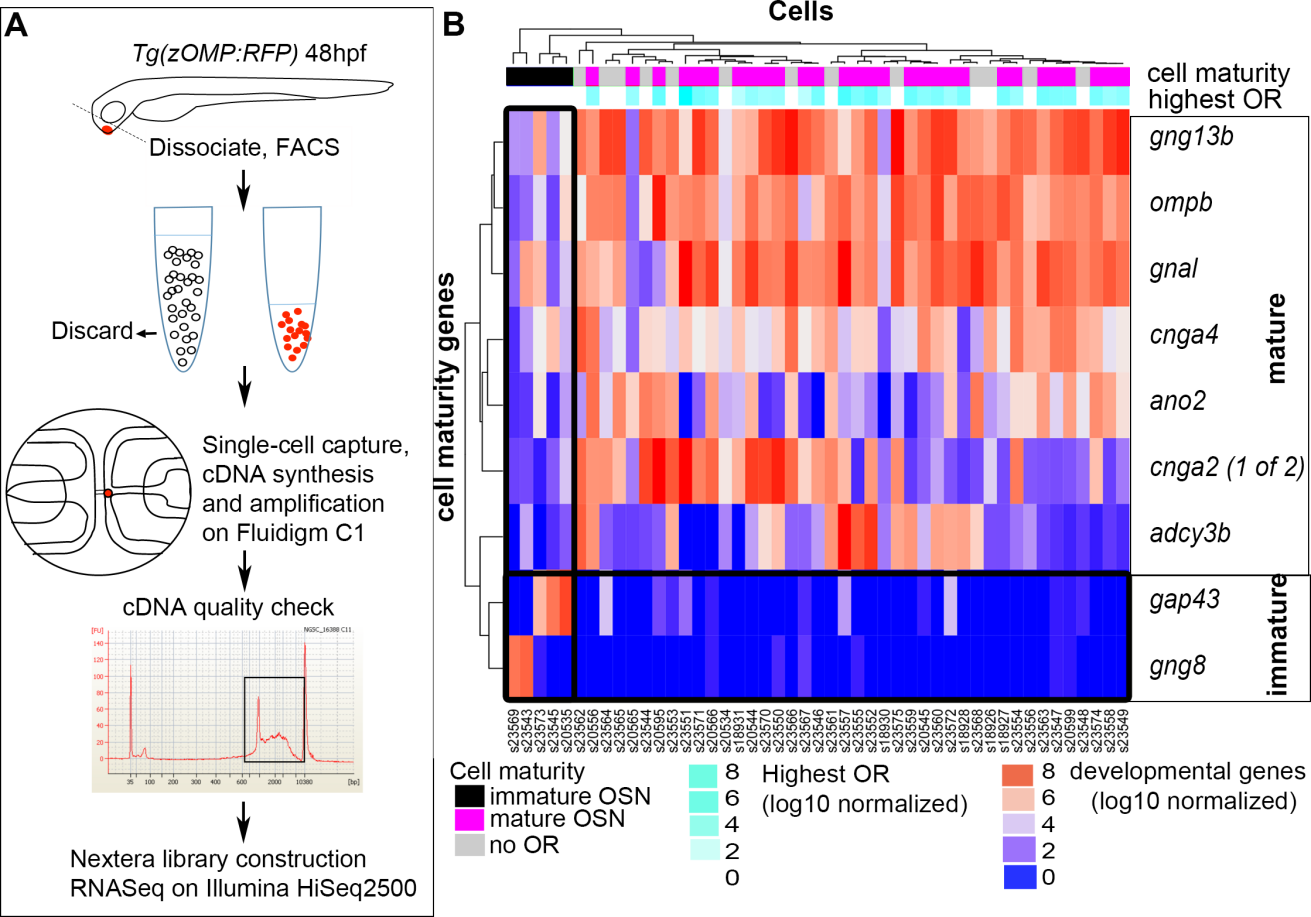
Isolation of single OSNs and identification of mature cells expressing a single predominant OR. (A) Method of isolation of single OSNs. Transgenic zebrafish embryos expressing OMP:RFP were raised to 48hpf. Olfactory epithelia were dissected and dissociated to obtain single-cell suspensions. OMP:RFP expressing neurons were enriched using FACS and loaded onto a Fluidigm C1 microfluidics chip to isolate single cells in singe wells. cDNA synthesis and amplification were carried out directly on the chip. Samples with high yields of cDNA and smooth distributions of RNA lengths were processed though Nextera library prep and Illumina sequencing. (B) A heatmap of normalized marker gene values for 47 single OSN transcriptomes. 5 cells were enriched in markers of progenitor and/or early mature stages (shaded in black on top bar). 42 cells showed strong expression of mature OSN marker genes and low expression of progenitor/earlier mature genes. High OR expression levels (second bar:cyan) were detected in 29 of these mature cells (shaded in magenta on top bar). Three color keys are shown at the bottom: the first pertains to cell age inferred in the topmost bar, the second pertains to log10 values of normalized (predominant) OR expression, and the third relates to log10 values of normalized developmental gene expression.

OSN axons first reach the bulb 24 hours post fertilization (24 hpf) and their initial targets, the protoglomeruli, are well formed by 72 hpf. OSNs from 48 hpf embryos were collected for analysis to maximize the chances of capturing transcripts relevant to axon guidance. We selected neurons expressing OMP, a marker of mature, axon bearing OSNs. Fish containing the zebrafish OMP promoter driven RFP transgene (*zOMP*:*RFP*; [37]) were intercrossed to obtain OMP:RFP expressing embryos (Fig 1A). RFP positive cells were isolated from dissociated olfactory epithelia by FACS. A cell suspension of the selected cells was loaded onto a Fluidigm C1 chip. Cells were sorted into individual wells, inspected visually to ensure that wells contained single cells, and cDNA was generated and amplified in each well. Fifty-eight single-cell cDNA samples had high yield and an even distribution of transcripts between 800bp-4kb as assessed using a Bioanalyzer. These samples were used for multiplex library preparation. Each sample was deep sequenced to a depth of 50 million reads to maximize the chances of capturing transcript differences in less highly expressed genes that may drive subpopulation fate or affect axon guidance. Reads were mapped to zebrafish genome assembly GRCz10 and to a spike-in RNA set. For most cells, approximately 50% of the reads mapped to the zebrafish genome while the remaining reads mapped to spike-in RNAs. In 8 cells less than 15% of the reads mapped to spike-ins. These were likely to be cell clumps that escaped our visual check and were excluded from further analysis. The 50 remaining single-cell transcriptomes typically had an average of 22.6 million mapped reads to the zebrafish genome per single-cell transcriptome. 2 cells had less than 3 million reads mapped to the zebrafish genome and were excluded from the dataset. A third cell was excluded because it had a disproportionate number of reads to a small number of genes.

### The majority of single cell OSN transcriptomes express late stage markers and a single predominant OR transcript

It was first determined if the remaining isolated cells varied in their maturity. Unsupervised hierarchical clustering was conducted based on the expression of OMP and other molecular markers for mature OSNs: *gng13b*, *gnal*, *ompb*, *cnga2*, *cnga4*, *ano2*, and *adcy3b* [31, 33]; and markers for immature OSNs or their precursors: *gng8* [33] and *gap43* [31, 33]. Five cells showed high expression of the immature markers, *gng8* and *gap43*, along with low expression of mature cell markers (Fig 1B: topmost bar, black shading). 42 cells showed minimal expression of early stage markers and strong expression of mature cell markers like *gng13b*, *gnal*, *ompb and cnga4*. Thirteen of these mature cells did not express appreciable ORs (Fig 1B: topmost bar, grey shading). They may have yet to turn on their expression of OR genes, or instead, they could be non-OR expressing OMP neurons. None of these cells were found to express another major category of olfactory receptors, the TAAR receptors. They were excluded from further analysis. The remaining 29 mature cells expressed OR transcripts, typically with a single one predominating (Fig 1B: topmost bar, magenta shading), and these were the cells used in all subsequent analyses. None of the immature cells expressed appreciable levels of OR transcripts. Thus, a single OR transcript is chosen very early in the development of zebrafish OSNs as the axons are making crucial targeting decisions.

### Classification of OSNs based on their predominantly expressed OR

**Fig 2:**
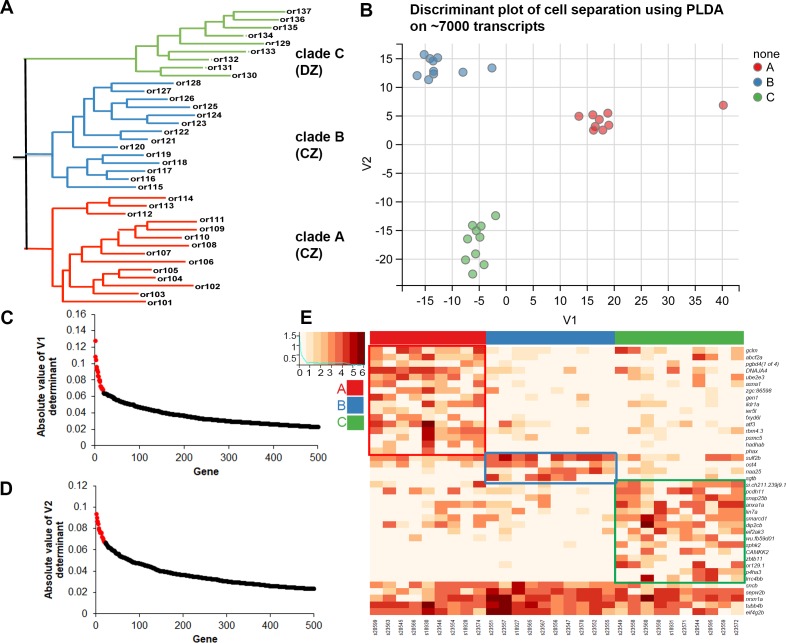
OSN transcriptomes can be differentiated into 3 categories according to the OR clade of their predominant OR. (A) Cladogram showing the 37 zebrafish OR sub-families organized into three major clades provisionally identified as clade A, clade B, and clade C. (B) PLDA was used to identify a combination of genes that provides the most useful classifiers for discriminating between the three clade-specific classes of OSNs. Two discriminant axes were extracted for the three classes that segregate cells into distinct clusters with no overlap. (C, D) The absolute values of co-efficients for the V1 and V2 determinants were sorted and plotted to reveal the most discriminative genes. Genes colored in red dots contributed the most toward the discrete clustering of cells and were used to make the heatmap in E. (E) Heatmap of normalized expression values of the top 40 ranked genes identified using pLDA. Red, blue, and green boxes indicate higher expression of genes in single OSNs expressing ORs from clade A, B, or C cell categories, respectively. Genes at the bottom are expressed in more than one clade of OSNs and are not boxed. Red, blue, and green bars at the top of the heatmap indicate clade-specific cell categories based upon their predominantly expressed OR.

OSNs that express closely related ORs have been shown to project to the same protoglomerulus [36]. The molecular homology between zebrafish ORs suggests a phylogenetic organization into three major clades that we have designated clades A, B, and C (Fig 2A, [4]). Clade A is comprised of subfamilies or101-114, clade B of subfamilies or115-128, and clade C of subfamilies or129-137. Experiments with BAC reporter constructs for 16 ORs from clades A and B labeled axons that projected to the CZ protoglomerulus, while those for 3 ORs from clade C projected to the DZ protoglomerulus [36]. We therefore classified single OSN transcriptomes by the expression of their predominant OR and assigned each to one of three OR homology clades: A, B, or C (Table 1). Nine cells were assigned to the clade A category, 10 cells to clade B category, and 10 to clade C category (Table 1). Cell s20599 (marked with *, Table 1) had less than a two-fold expression difference between or106-8 and or111-2. Since both these ORs belong to clade A, s20599 was assigned to clade A. Similarly, s23558 (marked with *, Table 1) had comparable expression levels for 2 OR transcripts, or133-2 and or129-1, both of which belong to homology clade C. Hence, it was assigned to the clade C category. It has been proposed that particular OR genes are first expressed at specific time-points during development [38–40]. Only OSNs that expressed markers indicative of mature OSNs were included in our analysis, and ORs from all three clades were equally represented in our dataset. It is possible that OSNs expressing particular ORs differentiated before or after others, but it is unlikely that the differences we see among OSNs categorized by clade can be ascribed to differing levels of maturity. For all subsequent analyses, we compared the expression of transcripts among cells categorized by clade (OR clades A, B, or C), with the underlying assumption that OR clade choice and axon targeting are closely related.

**Table 1:** OR transcripts detected in single OSN transcriptomes.

Sample	OR transcripts detected	OR Read Counts (per million mapped reads)	Predominant OR fold expression	Clade Assigned
**s18928**	**106–8;** 109–4; 109–7	**14123**; 180; 16	>78	A
**s18930**	**111–11;** 106–8	**55**; 2	>21	A
**s20545**	107–1	3835	>1000	A
**s20566**	**106–11;** 125–4	**8655**; 1	>5500	A
s20599*	106–8; 111–2; 111–11; 110–2	9160; 5172; 685; 299	>1.5	A
**s23546**	**112–1;** 103–1	**12914**; 2	>6700	A
**s23554**	**106–3;** 106–2; 132–5	**1647**; 6; 2	>270	A
**s23563**	**112–1;** 111–3; 103–4; 128–10	**14853**; 2; 1; 1	>5500	A
**s23574**	**111–4;** 111–11; 111–6; 111–3	**11205**; 1770; 1579; 4	>6	A
**s18927**	**121-2p;** 111–11; 106–8	**5768**; 9; 4	>600	B
**s20556**	**125–4;** 133–8; 106–7; 133–7	**5153**; 144; 49; 5	>30	B
**s20565**	115–5	**1037**	>1000	B
**s23547**	**128–10;** 112–1; 132–5; zgc:152857/119-1	**10143**; 3; 2; 1	>2800	B
**s23551**	**125–4;** 112–1	**24371**; 3	>6900	B
**s23552**	**zgc:152857/119-1;** 119–2	**31586**; 1	>30000	B
**s23555**	**125–5;** 125–7; 125–6; 125–3	**8376**; 207; 8; 1	>40	B
**s23557**	**119–2;** 132–5	**6548**; 5	>1300	B
**s23567**	**115–12;** 133–2	**98;** 1	>70	B
**s23570**	**116–1;** 111–4; 133–2	**706;** 3; 1	>200	B
**s18931**	**130–1;** 106–8	**154;** 3	>40	C
**s20544**	**129–1**	**3190**	>1000	C
**s20595**	**136–1;** 106–7; 133–1; 125–4; 106–8	**10114;** 676; 2; 2; 1	>14	C
**s23549**	**129–1**	**3483**	>3000	C
**s23550**	**136–1**	**788**	>700	C
s23558*	133–2; 129–1; 112–1; 132–5	2126; 1747; 2; 1	>1	C
**s23559**	**132–5;** 132–4	**31328;** 1	>2100	C
**s23560**	**134–1**; 132–5; 112–1	**773;** 5; 1	>160	C
**s23571**	**133–2;** 133–1; 133–5	**20600;** 9; 1	>2280	C
**s23572**	**129–1;** 133–2; 111–4	**1162;** 2; 1	>530	C

Predominant ORs and their read counts are highlighted in bold. Fold expression was computed by comparing the highest two ORs expressed in each cell.

The OR homology clade–A, B or C—to which each cell was assigned was determined based on the clade of the predominant OR.

Cells highlighted with * did not have a strongly predominant OR.

### Identification of clade-related marker genes

We first sought to identify OR clade-specific gene expression patterns in order to identify potential marker genes that could be used in the future to label each subpopulation of neurons. A total of 29,033 distinct mapped transcripts were filtered to identify those detected in 3 or more mature cells at a level of 100 or more raw counts. 7,027 transcripts met these criteria. Rather than computing gene-by-gene differential expression, we considered multi-variate combinations that might arise from coordinated differential expression of gene sets. We utilized Penalized Linear Discriminant Analysis (PLDA) as implemented in the Penalized LDA package [41], with a lasso penalty on the discriminant vector to induce feature scarcity. Applying this approach to high-dimensional gene expression covariation matrices effectively constrains the solutions when the number of variables is much higher than the number of classes [41]. We extracted two discriminant axes that separated the cells between the three OR clades (Fig 2B). The PLDA functions of these two axes contain 2886 genes. The absolute values of loading coefficients for each gene are displayed in rank order for the top 500 genes in each discriminant (Fig 2C and 2D). The 20 highest values for each discriminant axis are colored in red. To mitigate over-fitting the model, 40 genes, representing the cumulative top 20 loadings from each discriminant axis were used to re-compute the penalized LDA function. Cross-validation accuracy of this reduced discriminant function was 98.6%. The utility of this sparse feature set of 40 genes in capturing clade-specific structure within single cell OSNs transcriptomic landscape can be seen in Fig 2E. Genes whose expression is high in each clade related group are boxed. Genes *gclm*, *abcf2a*, *pgbd4*, *DNAJA4*, *ube2e3*, *asna1*, *zgc*:*86598*; *gen1*, *ildr1a*, *ier5l*, *fxyd6l atf3*, *rbm4*.3, *psmc5*, *hadhab*, and *phax* are strongly expressed in clade A cells. Genes *sulf2b*, *ost4*, *naa25*, and *sgtb* show elevated expression in clade B cells; while clade C cells show elevated expression for *si*:*ch211-239j9*.*1*, *pcdh11*, *snap25b*, *anxa1a*, *lin7a smarcd1*, *dlp2cb*, *eif2ak3*, *wu*:*fb59d01*, *sphk2*, *CAMKK2*, *zbtb11*, *or129-1*, *p4ha3 and lrrc4bb*. A majority of these genes encode intracellular metabolic enzymes, a smaller number are cell surface proteins, and one is a transcription factor. Some of these genes may serve as useful markers for clade-specific subsets of OSNs. Four of these genes are cell surface receptors and three of them are candidates for guidance genes: *ildr1a*, *pcdh11*, and *lrrc4b*. Interestingly, clade B cells may have a transcriptional profile that is distinct from that of clade A. This raises the possibility that OSNs expressing ORs from clade A as compared to clade B are distinct, even though they all project to the CZ protoglomerulus. The remaining genes are expressed in quantitatively but not qualitatively useful patterns, and are unlikely to be useful in classifying OSN subsets. Overall, our findings suggest that PLDA can identify genes that are expressed in OSNs that express ORs from particular clades.

### Identification of clade-related candidate guidance genes

Our primary focus was to identify candidate guidance-related genes that could account for the differential targeting of OSN axons to different protoglomeruli in the bulb. Target-P [42] was used to identify genes with N-terminal signal sequences. Signal sequences mark genes destined for intracellular compartments, the cell surface, or secretion. Since axon guidance receptors are associated with the cell surface and axon guidance cues are either associated with the cell surface, the ECM, or are secreted, this approach provides us with an unbiased gene set potentially involved in the guidance of olfactory axons. Target-P identified 6696 genes in the zebrafish transcriptome with an N-terminal signal sequence, and of these, 6197 were expressed in our single cell transcriptomes. Of these, 1156 genes were expressed in three or more mature cells at minimum raw counts of 100. OR genes were removed from the list.

**Fig 3:**
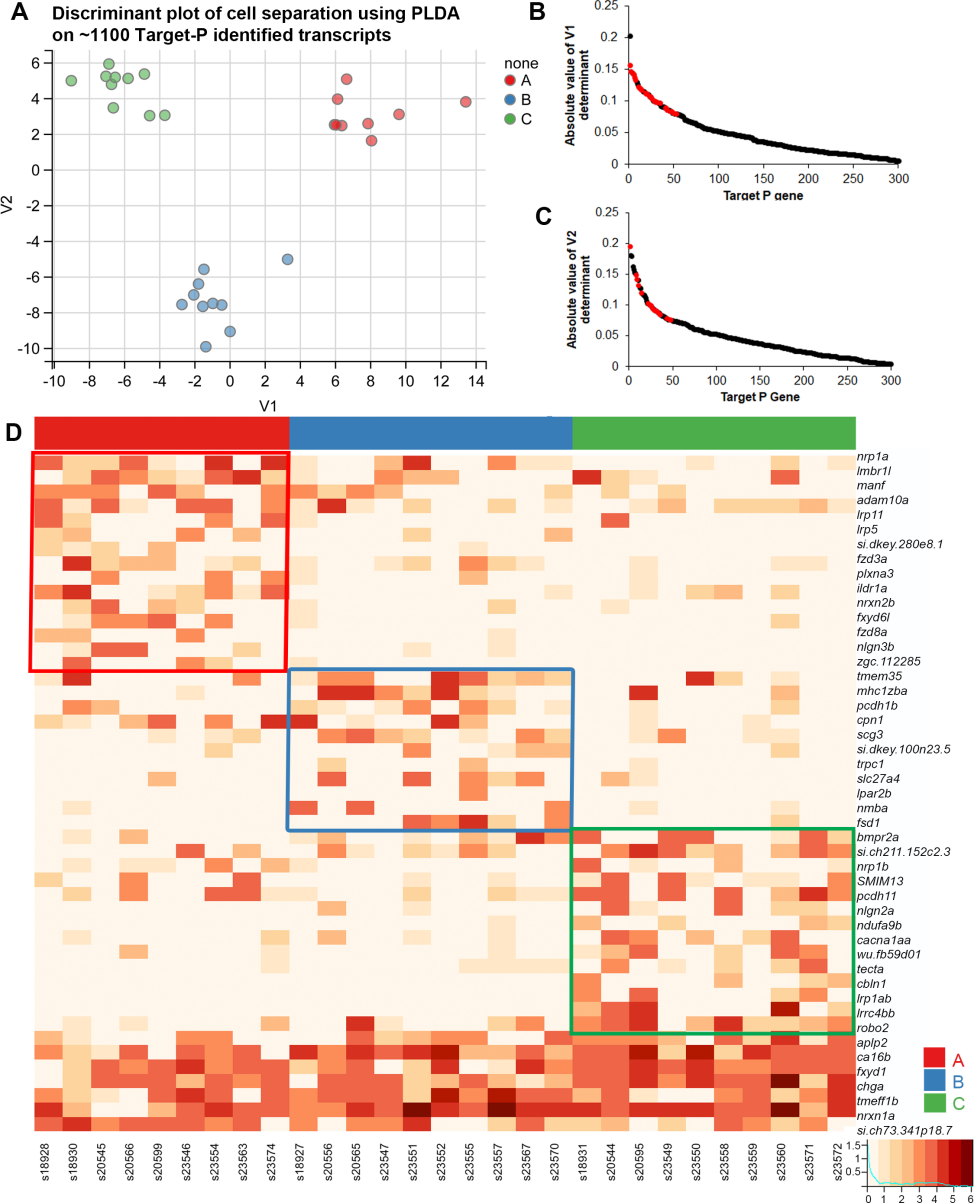
OR clade choice is related to the expression of distinct guidance gene transcripts. (A) PLDA using a ‘membrane-associated’ subset of transcripts selected using TargetP. OSNs can be discriminated into three distinct clusters along the two extracted determinants. (B, C) Distribution of the V1 and V2 loading values for 343 genes shows the top candidate plasma membrane associated or secreted transcripts (red dots) that contribute towards OSN discrimination. Top genes that have not been shaded in red localize to intracellular compartments like the endoplasmic reticulum, Golgi complex or lysosomes and hence are not likely to place a direct role in axon guidance. (D) Heatmap of normalized expression values of top 47 candidate guidance related transcripts. Red, blue, and green boxes indicate higher expression of genes in single OSNs expressing ORs from clade A, B or C, respectively. Genes at the bottom are expressed in more than one clade of OSNs and are not boxed.

Using PLDA, we extracted two discriminant axes separating cells of the three OR-clades. The PLDA functions contain 343 genes with signal sequences (Fig 3A). The genes loading to the V1 and V2 discriminant axes were plotted in descending order of their absolute value (Fig 3B and 3C). Sparse discriminant functions (see methods) from this feature set had cross-validation accuracy of 100%. Of the approximately top 50 loadings for each discriminant axis (105 total), we selected genes most likely to be secreted or localized to the cell surface in order to enrich for potential axon guidance-related molecules (Fig 3B and 3C, red dots). A total of 47 of 105 genes were selected as having the potential to be guidance related based upon annotations in UNIPROT [43]. Three of these are secreted and the rest are localized to the plasma membrane. Visualizing the expression of these 47 candidate ‘guidance genes’ in a heatmap shows that 40 of them are expressed most highly in just one of the three clade-related categories (Fig 3D). Some of the cell surface genes are canonical guidance receptors such as *robo2*, *nrp1a*, and *nrp1b*. *lrp5* also plays a role in axon guidance [44] while other genes like *nlgn2a*, *nlgn3b*, and *lrrc4b* have been shown to be important for synapse formation [45, 46]. These genes are novel candidates for playing a role in the guidance of OSNs. An interesting observation is that of the 47 guidance candidates, many belong to common families such as *nrp*, *lrp*, *fxyd*, and *fzd*. There are multiple instances where genes belonging to the same family show higher expression in different classes of neurons. For example, *nrp1a* has higher expression in clade A cells while *nrp1b* is has higher expression in clade C cells. *lrp11* and *lrp5* show higher expression in clade A cells while *lrp1ab* is highly expressed in clade C cells. On the other hand, *fzd3a* and *fzd8a* both show higher expression in clade A OSNs. These findings are consistent with the idea that a large number of signaling systems potentially contribute to OSN axon targeting, and that particular family members may guide single subsets of OSNs to their targets.

### Identification of clade-related transcription factors

**Fig 4:**
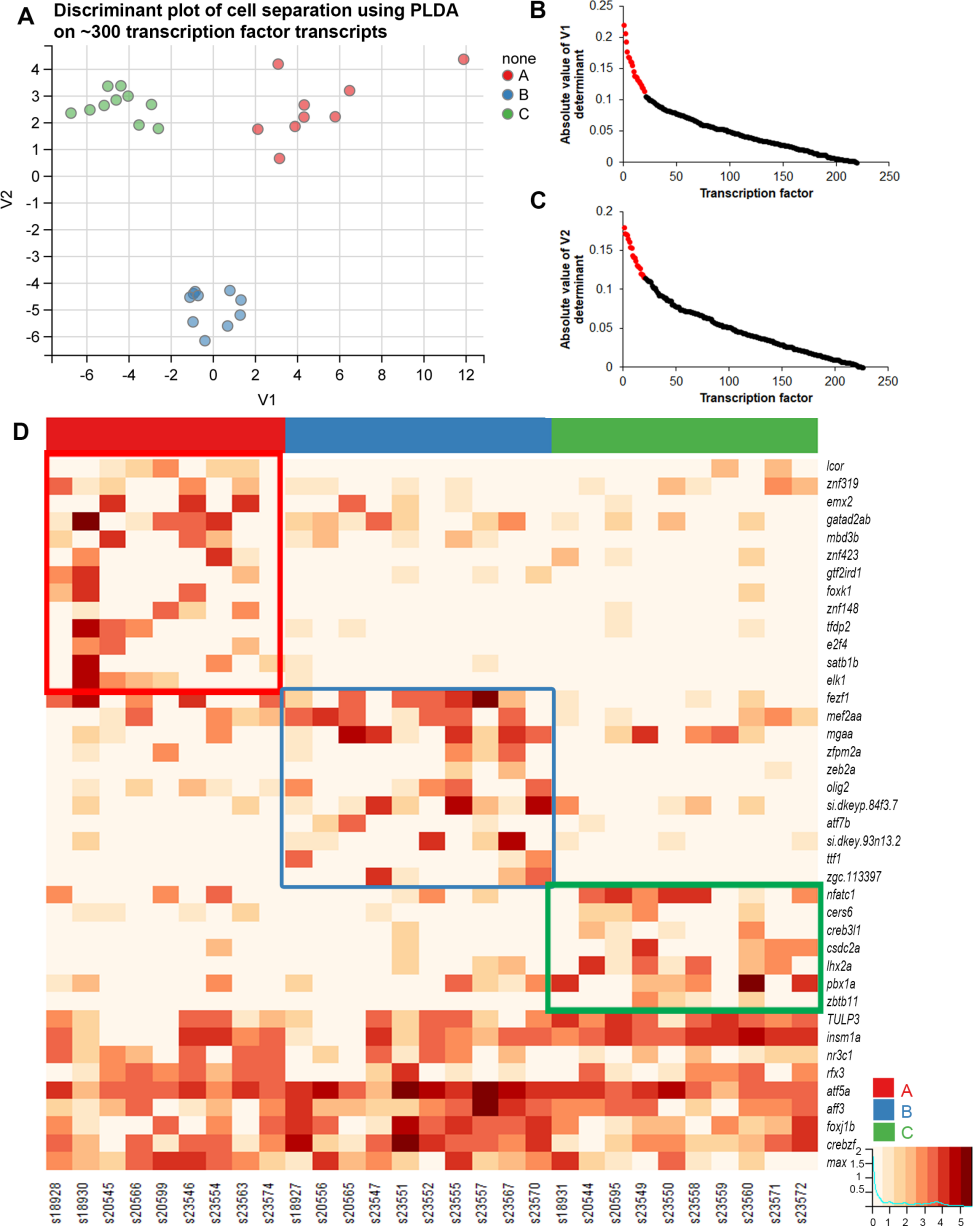
OR clade choice is related to the expression of distinct transcription factor transcripts. (A) PLDA using the transcription factor subset of transcripts. OSNs can be discriminated into three distinct clusters along the two extracted determinants. (B, C) Distribution of the loading values for 283 genes shows the top transcripts (red dots) that contribute towards OSN discrimination. (D) Heatmap of top 40 transcription factors boxed in red, blue, or green to highlight genes with higher expression in clades A, B, or C OSNs, respectively.

**Fig 5:**
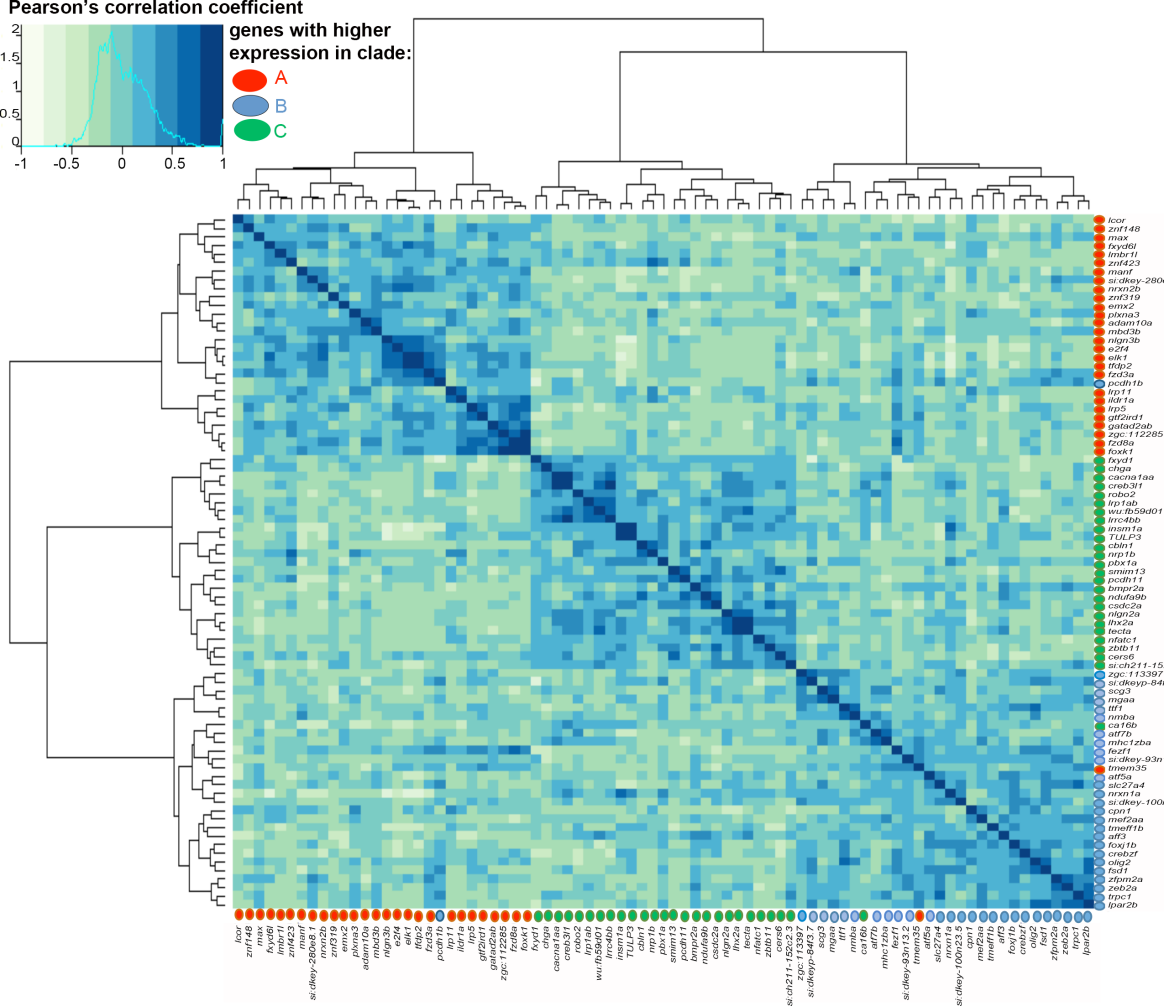
Clade specific co-expression of selected transcription factors and guidance related transcripts. Unsupervised clustering using average linkage of the Pearson’s correlation coefficients relating expression levels of 71 guidance related genes and transcription factors. Three distinct clusters are seen and each cluster is comprised of genes that show higher expression in the same clade as compared to the others. Transcripts expressed at higher levels in clades A, B, and C are indicated using red, blue, and green circles, respectively.

One mechanism that could explain the coordination of OR choice and OSN targeting would be that their expression is regulated by some of the same transcription factors. If true, there should be distinct transcription factor profiles associated with each clade-specific category of OSN. Of the 2345 annotated transcription factors in zebrafish (AnimalTFDB 2.0 [47]), 1610 were detected in our single cell dataset, 283 of which met the 3 cells with a minimum of 100 raw counts expression threshold criteria. PLDA using these transcription factors revealed 218 genes whose expression discriminated between the 3 clade-based categories of OSNs (Fig 4A, 4B and 4C). The top 20 genes based on the absolute value of the loadings in each discriminant axis were used to re-compute a penalized LDA function consisting of a sparse 40-gene feature set. Cross-validation accuracy of this sparse PLDA function was 95.9%. An analysis of these 40 genes contributing to the V1 and V2 discriminant axes identified 31 transcription factors that show higher expression in one of the 3 clade related categories of cells (Fig 4D). We next examined if the transcription factors and guidance genes identified through PLDA showed co-expression patterns correlated with the OR clades in which they were expressed. We computed Pearson’s correlation coefficients (Fig 5) between all pairs of a combined set of the top guidance related (from Fig 3D) and transcription related (from Fig 4C) transcripts that are expressed in only one clade-related category of OSNs. Genes are annotated for the clade-specific category in which they showed higher expression and ordered by unsupervised clustering using average linkage to produce the heat map in Fig 5 (clade A: red circles, clade B: blue circles, clade C: green circles). This correlation plot shows 3 easily identifiable blocks of higher correlation coefficients. The blocks correspond to OSNs expressing ORs from clades A, B, or C. These findings demonstrate the correlated co-expression of distinct ORs, transcription factors, and potential guidance related genes within our dataset.

### A subset of transgenically labeled OSNs that project to the CZ protoglomerulus conform to the clade A transcription profile

**Fig 6:**
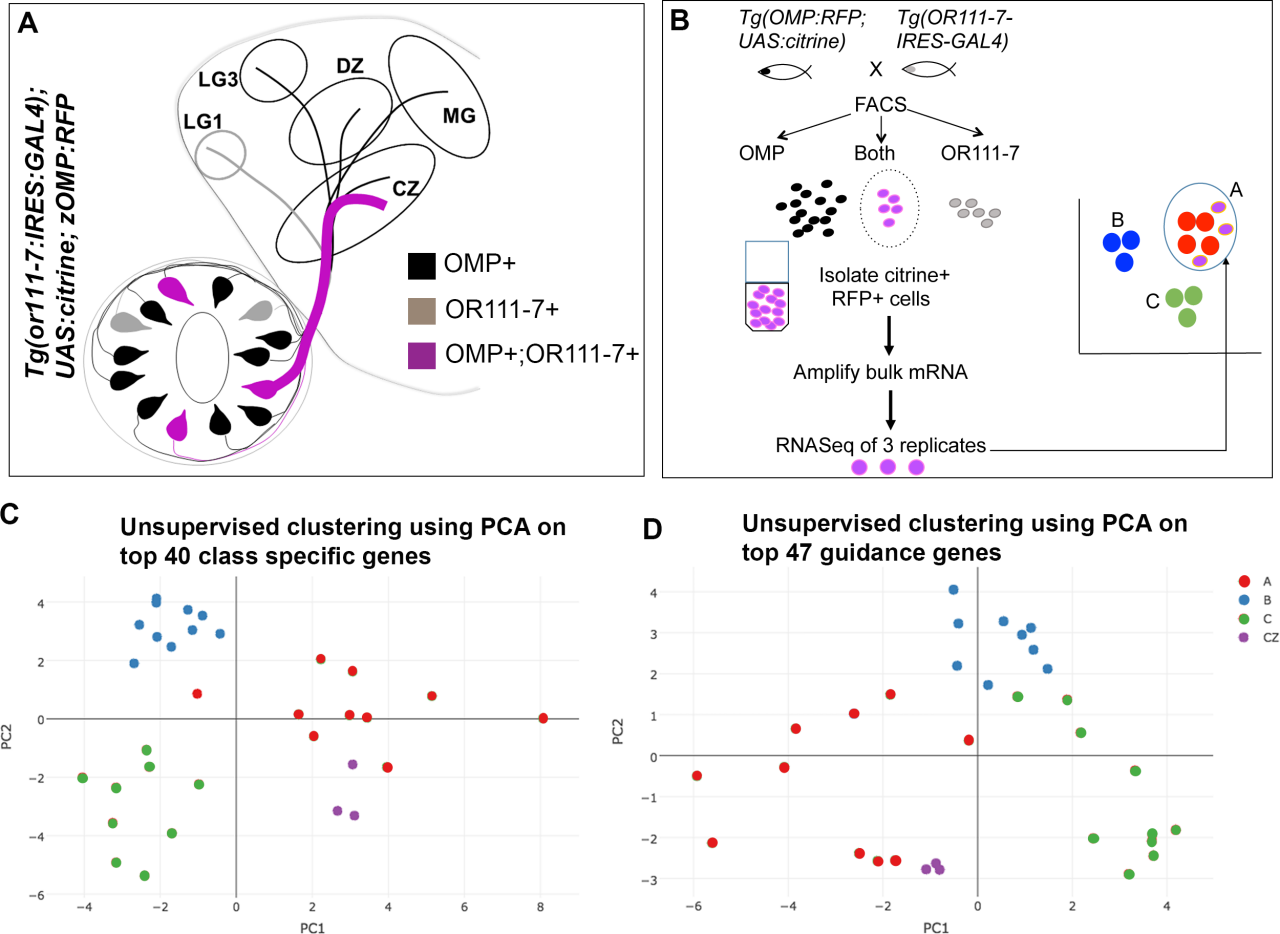
The transcriptome of a subpopulation of CZ-projecting OSNs that projects exclusively to the CZ protoglomerulus resembles those of clade A OSNs. (A) Schematic showing frontal view of a 72hpf double transgenic fish expressing *OMP*:*RFP* (black) and *or111-7*:*IRES*:*GAL4*; *UAS*:*Citrine* (purple and grey). Axons expressing both the transgenes are labelled in purple and project only to the CZ. CZ: central zone, DZ: dorsal zone, MG: medial glomerulus, LG1: lateral glomerulus 1, LG3: lateral glomerulus 3 (B) Method of isolation of *or111-7*:*IRES*:*GAL4* OSN population. Olfactory epithelia were dissected from double transgenic zebrafish embryos at 48hpf and dissociated into single cell suspensions. Two-way FACS was used to isolate cells that expressed both *OMP*:*RFP* (black dots) and *or111-7*:*IRES*:*GAL4;UAS*:*Citrine* (grey dots). This population (purple dots) was processed for RNA extraction and mRNA amplification. Three replicates were generated and compared with single OSN transcriptomes. (C) Unsupervised clustering with PCA of the *or111-7*:*IRES*:*GAL4* population and single OSNs using the top 40 class-specific transcripts shows clustering of *or111-7*:*IRES*:*GAL4* samples with clade A single cells. (D) Unsupervised clustering with PCA of the *or111-7*:*IRES*:*GAL4* population and single OSNs using the top 47 guidance related transcripts again shows clustering of *or111-7*:*IRES*:*GAL4* samples with clade A single cells.

We next wanted to evaluate the biological significance of the gene expression patterns identified through PLDA. We took advantage of the *Tg(or111-7*:*IRES*:*GAL4*) line of fish that carries a transgene in which *or111*-7 and *GAL4* expression are driven by the combined action of an enhancer element found near the *or111* subfamily gene cluster and the promoter for *or111*-7. The resulting expression of GAL4 labels a subset of OSNs whose axons project to the CZ and LG1 protoglomeruli ([48] Fig 6A, purple and grey axons). *or111*-7 is a member of the clade A category of ORs and the projection to CZ is expected while the projection to LG1 is aberrant [48]. We generated fish larvae containing *or111*-*7*:*IRES*:*GAL4*; z*OMP*:*RFP*; and *UAS*:*Citrine* transgenes. In these larvae OSNs expressing both Citrine and RFP project exclusively to the CZ protoglomerulus (Fig 6A, purple axons). 48 hpf olfactory epithelia were dissociated before performing a two-way FACS to select OSNs expressing both RFP and Citrine (Fig 6B). Approximately 1000 cells were collected for each of three replicates, total RNA was isolated, mRNAs were amplified by reverse transcription using oligo-dT fused to a T7 promoter sequence [49], libraries were constructed and sequenced. We hypothesized that the gene expression profile of *or111-7*:*IRES*:*GAL4*; *UAS*:*Citrine;* z*OMP*:*RFP* expressing OSNs should best match that of clade A single cells. Unsupervised clustering was performed with Principal Component Analysis using only the 40 class specific genes we identified (in Fig 2E) that most strongly discriminate between the clade A, B, and C categories of OSNs. The results are plotted for each of the 29 single cell transcriptomes together with the three replicates of the CZ projecting bulk transcriptomes (Fig 6C). First note that the single-cell transcriptomes organize into three distinct, clade-specific clusters on the two principal component axes. Second, the three CZ projecting OSN transcriptomes are most closely associated with the clade A cluster. The same result was obtained when only the 47 candidate guidance related genes identified by PLDA (in Fig 3D) were used to perform an unsupervised clustering of the single cell and CZ projecting transcriptomes (Fig 6D). This suggests that the guidance receptor transcriptome profile of the CZ projecting OSNs is most similar to clade A category OSNs. It provides additional support for the idea that the candidate guidance genes we identified are associated with CZ protoglomerular targeting.

### Validating guidance candidate expression patterns by fluorescent *in situ* hybridization (FISH)

**Fig 7:**
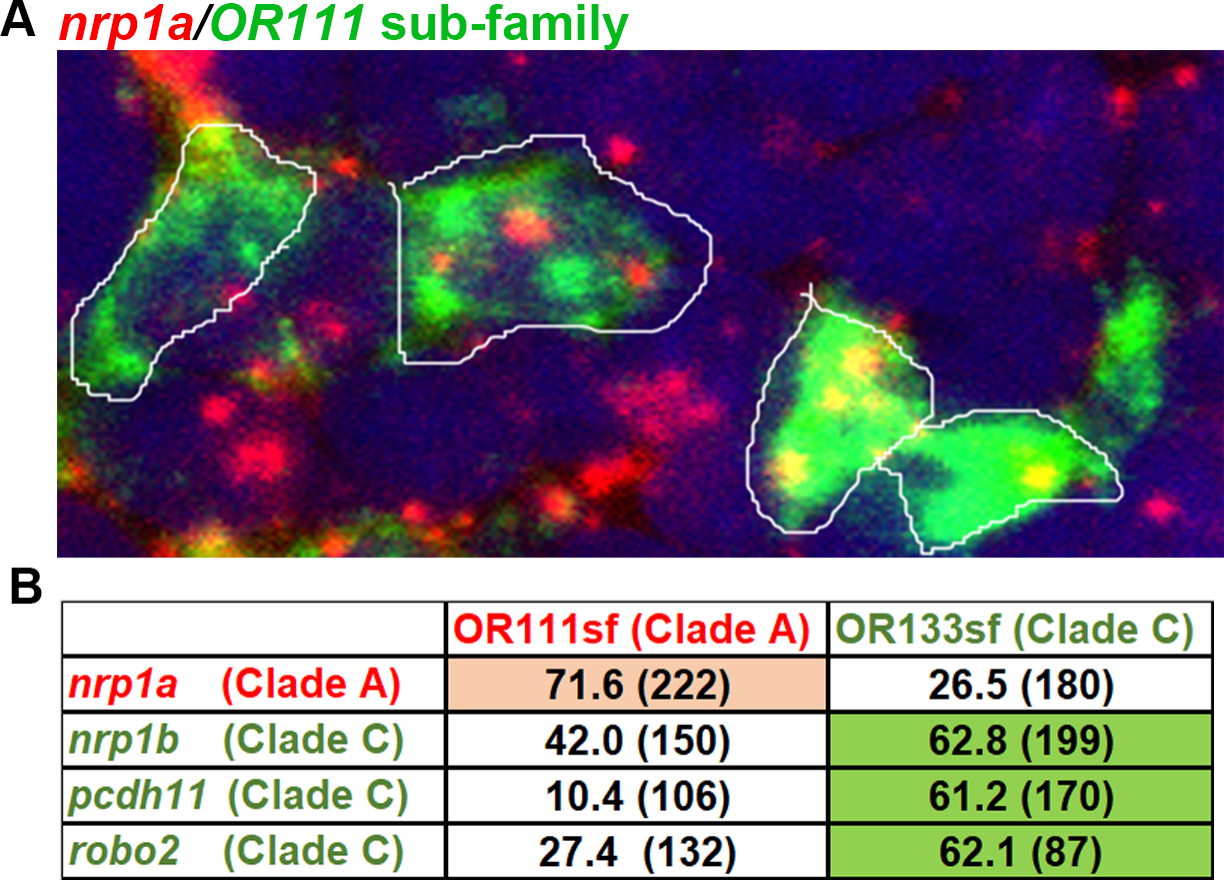
Confirmation of differential gene expression using dual fluorescent *in situ* hybridization. (A) Dual-FISH of *nrp1a* with an *or111* sub-family probe cocktail. Two cells have been highlighted to show that *nrp1a* signal (red) is localized in cells with *or111* subfamily labelling (green). (B) Summary of dual-FISH expression for four candidate guidance receptors. The percentage of OSNs expressing the indicated OR subfamily that also expressed the indicated candidate guidance transcript are noted. The total number of OR subfamily expressing cells counted are shown in parentheses. Pink shading indicates transcripts that showed higher overlap with the *or111* sub-family probe-mix and green shading indicates transcripts that showed higher overlap with *or133* sub-family probe mix. *nrp1a* shows greater overlap with *or111* subfamily expression than with *or133* subfamily expression. *nrp1b*, *pcdh11*, and *robo2* show greater overlap with *or133* subfamily expression than with *or111* subfamily expression.

*In situ* hybridizations were performed to verify that the expression of selected guidance-related genes is associated with specific sub-families of ORs. Double fluorescent ISH for each one of the four different guidance receptors *nrp1a*, *nrp1b*, *robo2*, or *pcdh11;* were performed with sub-family (sf) specific cocktails of probes for the *or111*sf (clade A) or the *or133*sf (clade C). Cells labeled with a particular subfamily of OR probes were visually inspected for the presence or absence of guidance gene signal (Fig 7A). Based on our single-cell transcriptome profiles, we would expect clade A enriched gene expression to overlap with *or111*sf expression, while clade C enriched gene expression to overlap with that of *or133*sf expression. As expected, *nrp1a* is co-expressed to a greater degree with *or111*sf than with *or133*sf expressing OSNs (Fig 7B). Also as expected, *nrp1b*, *robo2*, and *pcdh11* were co-expressed to a greater degree in *or133*sf as compared to *or111*sf expressing OSNs. Thus, the pattern of association of guidance receptors with OR subfamilies as seen by FISH is consistent with the single-cell RNA-seq approach. It is important to note that the overlap is a qualitative rather than a quantitative measure and may not reflect true differences in expression levels.

### Testing selected guidance candidates for their contribution to OSN targeting

**Fig 8:**
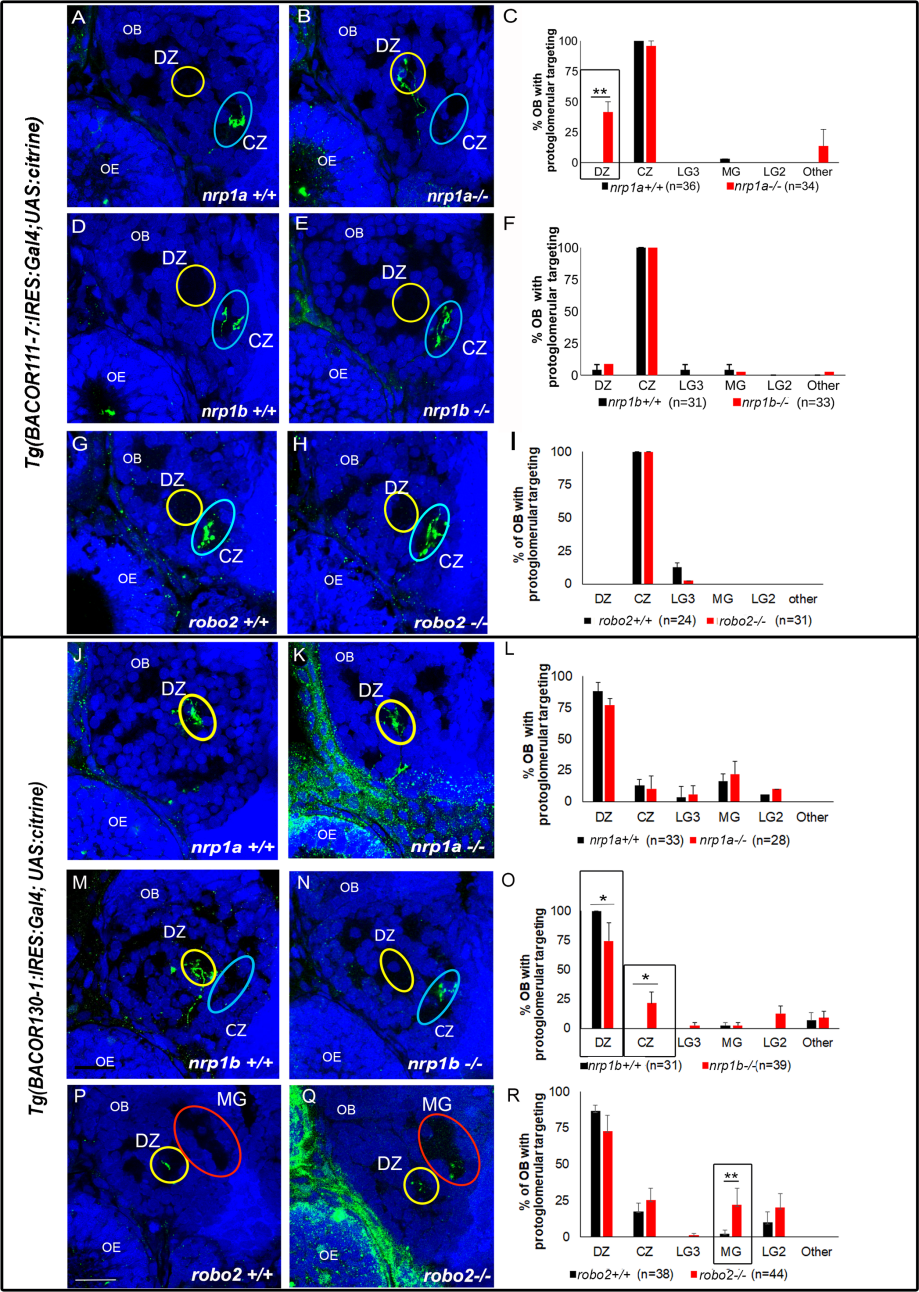
*Nrp1b* and *robo2*, but not *nrp1a*, are required for axonal targeting to the DZ. *Tg(BACor111*-7:*IRES*:*GAL4; UAS*:*citrine*) (‘*or111-7’)* expressing OSNs project axons to the CZ in wild-type olfactory bulbs (A, D, G) while *Tg(BACor130-1*:*IRES*:*GAL4; UAS*:*citrine*) (‘*or130-1’)* expressing OSNs project axons to the DZ in wild type olfactory bulbs at 72 hpf (J, M, P). In *nrp1a-/-* larvae, *or111*-*7* axons misproject to the DZ (B, C). Mistargeting of *or111-7* axons is not observed in *nrp1b-/-* (E,F) or *robo2-/-* larvae. (H, I). However, in *nrp1b-/-* larvae, *or130-1* axons misproject to the CZ (N, O). Similarly, *robo2-/-* larvae show misprojections of *or130-1* axons to the MG (Q, R). Mistargeting of *or130-1* axons is not observed in *nrp1a-/-* larvae (K,L). Fisher’s exact test (two-tailed) * = p<0.05, ** = p< = 0.005. CZ: central zone, DZ: dorsal zone, MG: medial glomerulus, LG2: lateral glomerulus 2, LG3: lateral glomerulus 3. OE: olfactory epithelium, OB: olfactory bulb.

In order to test if the candidate genes identified by sc-RNA-seq have a functional role in axon guidance, we used OR reporter lines that express both an OR and the GAL4 activator directly under the control of the OR promoter. *Tg(BACor111*-7:*IRES*:*GAL4)* expresses GAL4 in CZ-projecting OSNs that belong to clade A OSNs, and *(TgBACor130*-1:*IRES*:*GAL4)* expresses GAL4 in DZ-projecting clade C OSNs. GAL4 expressing axons are visualized when these lines are crossed to a transgenic *UAS*:*Citrine* reporter line [36]. We first asked if *nrp1a* is required for the targeting of either of these axon populations. Our data shows that *nrp1a* transcripts are highly enriched in clade A cells which are known to project to the CZ, and further, that *or111-7*:*IRES*:*GAL4* expressing OSNs have a similar expression profile as clade A OSNs. In contrast, *nrp1a* is not highly expressed in clade C sensory neurons. We therefore predict that *nrp1a* should be required for the correct targeting of *or111*-7 axons but dispensable for the targeting of *or130-1* axons. The point mutant *nrp1a*^*sa1485*^ has a nonsense mutation at aa206 which produces a premature stop in Nrp1a [50]. Either *Tg(nrp1a*+/-; *BACor111-7*:*IRES*:*GAL4*) or *Tg(nrp1a*+/-; *BACor130-1*:*IRES*:*GAL4)* fish were crossed with *Tg(nrp1a*+/-; *UAS*:*Citrine)* fish and their progeny genotyped and analyzed. Consistent with expectations, *or111-7* expressing, citrine labeled neurons that should project exclusively to the CZ protoglomerulus, show significantly higher ectopic misprojections to the DZ in *nrp1a* -/- mutant as compared to their WT siblings (Fig 8A–8C). In contrast, *or130-1* expressing neurons navigate normally to the DZ in *nrp1a* -/- mutants (Fig 8J–8L).

We next tested the contribution that *nrp1b* makes to the targeting of OSNs expressing either *or111-7* or *or130-1*. *nrp1b* might be expected to contribute to DZ targeting as it is more highly expressed in clade C as compared to other OSNs. *nrp1b*^*fh278*^ carries a nonsense mutation at aa116 that leads to the generation of truncated protein [50]. WT and mutant larvae were generated as described above for analysis. As predicted, *or130-1* expressing OSNs that should project exclusively to the DZ protoglomerulus have significantly more ectopic CZ misprojections in *nrp1b*-/- mutants as compared to their WT siblings (Fig 8M–8O). In contrast, *or111-7* neurons projected without additional errors to the CZ (Fig 8D–8F). Thus, *nrp1b* is required for normal axonal guidance of clade C but not clade A OSNs.

Lastly, the contribution of *robo2* to DZ protoglomerular targeting was tested. *robo2* is expressed more highly in clade C as compared to clade A OSNs. We employed the *robo2*^*ti272*^ mutation that carries a nonsense codon at aa635 and generates a truncated robo2 protein. This mutation has been shown to cause a dramatic misprojection phenotype in the retinal axon projection to the tectum [51]. WT and mutant larvae were generated as described above for analysis. Abnormal projections of *BACor130-1*:*IRES*:*GAL4* expressing OSNs were observed at a higher rate in *robo2* mutants as compared to WT larvae, particularly misprojections to the MG protoglomerulus (Fig 8P–8R). In contrast, there is no increase in mistargeting of CZ projecting *BACor111-7*:*IRES*:*GAL4* (clade A) expressing OSNs (Fig 8G–8I). These data are consistent with clade C OSNs, but not clade A OSNs, requiring *robo2* for their normal targeting to the DZ protoglomerulus.

In each of the three mutants we examined, OSNs expressing a candidate guidance molecule at a higher level required that candidate for normal axonal targeting. Those OSNs expressing the same candidate at a lower level did not require it for targeting. These results strongly suggest that the candidate genes identified by single cell RNA-seq are likely to play a role in guidance and targeting of the clade-specific OSNs in which they are enriched.

## Discussion

### Summary of findings

We have identified subsets of candidate axon guidance-related genes whose differential expression is related to the clade from which an OSN chooses its OR. We have confirmed that three of these candidates: *nrp1a*, *nrp1b*, *and robo2*, are functionally required for normal olfactory axon targeting in specific subsets of OSNs that express particular ORs. Not only axon guidance related genes, but also transcription factors, are differentially expressed in an OR clade-specific manner. These findings are consistent with a model in which distinct subsets of transcription factors coordinate general aspects of OR choice and axon targeting in OSNs.

### The suitability of using PLDA to identify differentially expressed genes

The dimension reduction technique, PLDA, has proven to be highly useful in this study for the identification of differentially expressed class-specific genes, guidance factors, and transcription factors. This approach has previously been successfully employed to characterize human brain cell types [52]. By focusing on a small number of genes that provide the most discriminative power for each gene set we analyzed, we have tried to avoid over-fitting problems that can arise with large multidimensional datasets. PLDA requires *a priori* knowledge of cell classification in order to maximize class separation using the available set of features. It would be useful in many studies where cells can be grouped based on molecular markers, developmental age or cellular state [53–55]. It is however not applicable to studies where there is no prior knowledge of sub-population characteristics and would be a poor choice to identify entirely novel cell sub-types. This method was suitable in our study since we were able to classify OSN transcriptomes into three categories based upon the predominant OR transcript expressed in each OSN. One caveat of using single cell RNA-seq datasets to identify differentially expressed genes is that the high frequency with which transcripts fail to be identified in any given cell can lead to the appearance of differentially expressed genes that arise solely from random fluctuations in transcript detection. Similarly, noise in the data could result in a failure to detect the actual differential expression of a gene. For these reasons, we have taken a conservative approach in identifying differentially expressed genes by choosing a limited number of genes with the highest coefficients of discrimination for each determinant. The weighted contribution of each gene to the discrimination between cell classes can be ranked and sorted to identify the most discriminating genes, although the cut-off for the most informative genes is somewhat arbitrary.

These considerations make it essential to employ tests of identified genes for true patterns of differential expression and/or their expected biological function. One test we performed was to use a pair of transgenes to isolate a population of OSNs that extend axons exclusively to the CZ protoglomerulus. The transcriptomes of these cells matched with reasonable fidelity clade A-specific marker genes as well as the guidance-related gene set for clade A OSNs. As all of the OR clade A expressing OSNs we have tested project to the CZ protoglomerulus [36], these findings provide a strong affirmation of the differential expression of these genes in CZ projecting OSNs. In a more direct approach, all four of the candidate genes chosen for FISH showed expression patterns that were consistent with the single-cell RNA-seq data. Finally, a test of biological function delivered the expected phenotypes for three candidate genes. The axons of OSNs in which each candidate was highly expressed projected aberrantly in mutants, while the axons of OSNs in which the candidate was expressed at a lower level projected normally. These results give us confidence that the guidance-related genes identified by PLDA in this study are good candidates for roles in OSN axon guidance.

### OSNs can be divided into three categories that express distinct transcription factors and guidance-related genes

A single cell approach has enabled us to characterize subsets of OSNs in the absence of any known molecular markers other than the ORs they express. We confirmed our working hypothesis that OSNs choosing to express ORs from each of the three clades of OR genes can be divided into three separate categories based on their differential expression of specific transcription factors, guidance related molecules, or other miscellaneous genes. It is interesting that clade A and B OSNs can be distinguished in this way, as both appear to project to the CZ protoglomerulus [36]. Future studies will be directed towards determining if clade A and B OSNs target different domains within the CZ. The CZ is the largest protoglomerulus at 72 hpf and is reported to split in two to form the maG and the vmG/vaG protoglomeruli by 96 hpf [56]. It will be of interest to determine whether these two protoglomeruli are differentially targeted by clade A and clade B OSNs. Although our findings support the OR clade-based categorization we imposed on OSNs, it does not preclude additional subcategories within each clade-specific category, or even alternative categories that cut across the clade-related categories.

### The specialized roles of *nrp1a* and *nrp1b* in olfactory axon targeting

We find that *nrp1a* is required in the zebrafish for the proper targeting of OSN axons to the CZ protoglomerulus, but not to the DZ protoglomerulus. In contrast, *nrp1b* is required for axons targeting the DZ, but not the CZ. As mentioned previously, semaphorins play important roles in organizing the olfactory projections in both the fly and mouse. In the fly, semaphorins organize the positioning of dendrites of projection neurons within the antennal lobe that serve as the targets for sensory axons, determine the ventromedial trajectory of OSN axons, and participate in glomerular targeting of sensory axons [10, 15, 57–59]. In the mouse, Sema3A helps to organize fasciculation of OSN axons in the olfactory nerve and contributes to the positioning of glomeruli in the bulb [60–63]. The clade and target specific requirement for *nrp1a* as compared to *nrp1b* observed for these two paralogs demonstrates that they have evolved to acquire independent functions in axon targeting in zebrafish. Whether this difference originates in their differential patterns of expression, or differences in their functional responses to ligands, is unknown. Previous work has shown that CZ projecting axons have an *nrp1a*-dependent repellent response that is mediated by *sema3d* expressed anteriorly in the bulb [64], but many other potential semaphorin ligands are expressed in the bulb and some of them could have additional guidance effects. The ligand(s) that mediate Nrp1b’s contribution to DZ targeting are unknown. Further defining the specific expression requirements and ligand responsive properties of these two neuropilins will provide an interesting perspective on the evolution and specialization of these very important signaling molecules.

### Robo2’s contribution to olfactory axon targeting

We find that *robo2* is required for the normal targeting of DZ but not CZ projecting axons. In the fly, slit/robo interactions are required for the normal dorsal-ventral positioning of glomeruli [65]. Similarly, Slit/Robo signaling is an important determinant of dorsal-ventral targeting of OSN axons in the mouse main olfactory bulb [11–12, 66]. In the accessory olfactory bulb, Robo2 is essential for basal vomeronasal sensory axons to specifically target the posterior compartment of the accessory olfactory bulb [67]. It is unclear whether our results are consistent with a shift along the dorsal ventral axis, or whether they represent the spreading of axons into a new compartment. To address this issue in the future, it will be necessary to identify the location at which pathfinding errors first occur and the source of the guidance signal that Robo2 is responding to. Robo2 has previously been observed to play a key role in olfactory axon guidance and entry into the olfactory bulb of fish [68]. Loss of *robo2* induced defasciculation of the ON, errant OSN axons failing to enter the bulb, impaired protoglomerular organization, and in the adult, mispositioned glomeruli. Miyasaka et al. suggested that some of these effects might be a consequence of the errors made by a group of early projecting pioneer axons in the mutant, and that later extending OSNs project abnormally because they follow the misprojecting pioneers. If there was any disruption of protoglomeruli in the *robo2* mutant larvae we examined, it was not sufficient to interfere with protoglomerular identification. Only occasionally did we detect BAC labeled sensory axons failing to enter the bulb (2/75 preparations). As we observed the specific perturbation of axon projections to the DZ but not the CZ, it is unlikely that the misguidance phenotype we observed was caused by general defasciculation of the ON. It is possible that Slit expression surrounding the ON helps to organize the selective fasciculation of Robo2 expressing axons within the nerve, just as Sema3A expressed in the tissues surrounding the mouse ON help to organize the selective fasciculation of Nrp1 expressing axons [61]. Alternatively, slits expressed within the telencephalon or in immediately surrounding tissues [68] may affect the trajectories of Robo2 expressing axons.

### A model for olfactory map formation

Our findings suggest that OSNs first project to a particular protoglomerulus depending upon the general category of OR they choose to express. Thus, OSNs expressing many different clade A ORs all project to the CZ protoglomerulus. This is in stark contrast to the adult olfactory bulb in which axons project to a much larger number of OR-specific glomeruli. Nevertheless, the early olfactory map is not formed at random. OSNs expressing the most closely related ORs specifically target the same protoglomerulus. We were able to identify clusters of transcription factors and guidance-related molecules that are differentially expressed between three OR clade-related subgroups of OSNs. We propose a model whereby the choice of clade from which an OR is expressed is coordinated with the expression of particular axon guidance receptors. We hypothesize that some of the differentially expressed transcription factors we have identified could help coordinate OR and guidance factor expression. This model makes the initial targets of OSN axons, the protoglomeruli, intermediate targets that are further subdivided and refined later in development as the axons of OSNs expressing a particular OR aggregate together and segregate from all others to form OR-specific glomeruli.

In conclusion, we have exploited the zebrafish model system and the power of single cell RNA-seq to characterize three distinct OSN subpopulations at a very early stage of olfactory system development. The guidance-related candidates we have identified likely play a crucial role in targeting specific axonal populations to their first targets in the olfactory bulb. Further studies are needed to characterize the physiological roles of the remaining candidate guidance genes in the formation of the early olfactory map. A second avenue of future work will be to explore whether and how the transcription factors we identified coordinate the expression of ORs and guidance related factors. Finally, the expression-based approach we took to identify important transcription and guidance factors should be generally applicable to many other developing systems.

## Materials and methods

### Ethics statement

Animal husbandry and procedures adhered to the guidelines prescribed by National Institutes of Health Guide for the Care and Use of Laboratory Animals. All animal studies were fully approved by the University of Pennsylvania Institutional Animal Care and Use Committee (IACUC) protocol numbers 804944 and 804895.

### Zebrafish maintenance and transgenic lines

Adult zebrafish were raised and maintained according to standard procedures [69]. Veterinary care was supervised by University Laboratory Animal Resources (ULAR) at the University of Pennsylvania. Larvae were staged based on hours post fertilization (hpf) and were raised at 28°C. *Tg(omp*:*lyn-RFP)* line was a gift from the Yoshihara laboratory at the RIKEN Brain Science Institute, Saitama, Japan [37]. The *Tg(or111-7*:*or111-7-IRES*:*GAL4)*, *Tg(omp*:*GAL4)* and *Tg(UAS*:*gap43-citrine)* lines were described by Lakhina et al [48]. *Tg(BACOR111-7*:*IRES*:*GAL4)* and *Tg(BACOR130-1*:*IRES*:*GAL4)* were described by Shao et al [36].

### Isolation of OMP expressing cells by FACS

Adult heterozygote fish carrying the z*OMP*:*RFP* transgene were crossed to each other to obtain embryos that expressed zOMP:RFP. Timed matings allowed us to follow the developmental stages. Embryos were bleached at 24hpf and allowed to develop till 48hpf. Chorions were removed by treatment with pronase at 24hpf. Embryos were anesthetized using tricain and dissected in Ringer’s solution to isolate the anterior half of the head containing the olfactory pits. Approximately 200 embryos were dissected for each FACs experiment. The olfactory pits were dissociated in 2.5% trypsin at 28°C for 30 min. At the end of incubation, the trypsin was neutralized using soy-trypsin inhibitor and the cell suspension was spun down in a clinical centrifuge at 2000rpm for 5 min. The pellet was resuspended in Hank’s Balanced Salt Solution (HBSS) buffer (Ca++/Mg++ free, without Phenol Red) with 1% BSA and 25mM HEPES. Cells were sorted based on forward scatter, side scatter and RFP fluorescence using BD FACS Aria II SORP, BD FACS Aria II U, or BD-Influx sorters (all BD Biosciences). Approximately 5500 OMP:RFP expressing cells were isolated for each of two independent experiments. For *OR111-7*:*IRES*:*GAL4* bulk cell isolation, adult heterozyogtes carrying z*OMP*:*RFP;UAS*:*citrine* were crossed to adult heterozygotes for *OR111-7*:*IRES*:*GAL4*. Embryos that expressed both *OMP*:RFP and *OR111-7*:*IRES*:GAL4;*UAS*:citrine were selected for sorting. 48hpf embryos were dissected as before except that cell suspensions were sorted based on GFP and RFP fluorescence. Approximately 1000 cells that expressed both were collected in each of 3 independent experiments.

### Single-cell capture and RNA-seq

Approximately 5000 OMP:RFP-expressing cells were loaded onto a Fluidigm-C1 microfluidics chip (Fluidigm) (size 5-10uM). Single cells were sorted into individual wells and visually inspected under a microscope for capture. 49 and 47 single cells, respectively were captured in two independent runs. Wells with no cells or more than one cell were marked to be discarded. cDNA synthesis and amplification was conducted using SMARTer amplification technology (Clontech). Fluidigm spike-ins or ERCC spike-ins were included in the reaction mix. cDNA yield was assessed using Agilent BioAnalyzer (Agilent Technologies, Santa Clara, CA). A minimum cDNA yield of 8ng per cell was required for library preparation. 20 cells from the first run and 38 cells from the second run passed this criterion. These were independently processed for pooled library preparation using Nextera XT DNA Library prep kit (Illumina). The resulting libraries were deep-sequenced on HiSeq2500 (Illumina) using the paired end 100-base pair protocol to a depth of 50 million reads per cell at the Next-Generation Sequencing Core of the Perelman School of Medicine, University of Pennsylvania.

### Read QC and mapping

Sequencing adapters, poly-A tails, and unknown nucleotides (Ns) were removed from the ends of reads using in-house trimming software. Additionally, low confidence nucleotides (Phred score less than 20) were treated as unknown and replaced with Ns. Reads shorter than 30bp after trimming were discarded. Resulting reads were aligned to the *Danio rerio* reference genome, build GRCz10, and to spike-in transcript sequences using STAR (Spliced Transcripts Alignment to a Reference) [70] aligner version 2.4.0. VERSE [71] running in Hierarchical Assign mode was used to assign mapped reads to gene features. The average number of mapped reads per cell was 55 million. Two of the 58 cells had less than 3 million reads mapped to the zebrafish genome and were discarded. The remaining 56 single cell samples had an average of 7,483 genes per cell with an average of 2,565 reads per gene. GEO accession id GSE103692. https://www.ncbi.nlm.nih.gov/geo/query/acc.cgi?acc=GSE103692

### Genome mining for genes with potential guidance-related activity and transcription factors

Zebrafish genes from GRCz10 version 80 were run on TargetP 1.1 server (Center for Biological Sequence Analysis, Technical University of Denmark http://www.cbs.dtu.dk/services/TargetP/) [42]. Genes with reliability class (RC) score = 1 were selected as containing a signal peptide and included in a list of candidate guidance genes. AnimalTFDB2.0 (Guo Lab, College of Life Science and Technology, HUST, China http://bioinfo.life.hust.edu.cn/AnimalTFDB/) [47] was used to download a list of zebrafish genes with known or putative transcription factor activity.

### Data thresholding and Penalized Linear Discriminant Analysis

The single-cell dataset of mature cells was filtered to include only those genes that were expressed in three or more cells at a minimum raw read count of 100. We identified linear combinations of genes that maximized between-class separation in high-dimensional gene expression covariation matrices (where the number of features exceeds the number of observations) using the ‘penalizedLDA’ R package version 1.1, which implements the Penalized LDA as originally conceived by Witten and Tibshirani [41]. A lasso penalization of 0.05 was applied to the feature coefficients in the discriminant vectors. Reduced discriminant functions were generated by using the top 20 genes ranked by the absolute value of their loading values in each discriminant axis and re-computing the Penalized LDA function using this sparse 40-gene feature set. Six-fold cross-validation was implemented via the ‘penalizedLDA’ package in R to test the accuracy of the ‘sparse’ 40-gene PLDA classifier, as well as to select the appropriate lambda tuning parameter value.

### Principal component analysis, correlation analysis and heatmaps

Top genes from single-cell PLDA analysis the using entire transcriptome or TargetP subset were used to compute a PCA function on single-cell and OR111-7-IRES-Gal4 bulk RNA-seq dataset using gene expression values that were DESeq2 normalized [72]. Correlation analysis of transcription factors and guidance genes was performed by computing the Pearson’s correlation co-efficients between genes followed by hierarchical clustering using average linkage. All heatmaps were plotted using ggplots package in PIVOT [73] using normalized gene expression values.

### Zebrafish mutants

*nrp1a*^*sa1485*^, *nrp1b*^*fh278*^ and *robo2*^*ti272*^ mutants have been described previously [51, 64]. These were crossed to *Tg(BACOR111-7-*:*RES*:*GAL4)*, *Tg(BACOR130-1*:*IRES*:*GAL4)*, or *Tg*(*UAS*:*citrine)* to generate adult heterozygotes that carried the appropriate transgenes. Heterozygous parents were mated to obtain fluorescent progeny that were collected at 72 hpf. Genomic DNA was extracted from tails and genotyped using PCR for *nrp1b* (described in [64]) and *robo2* (described in [74]) or KASP assay for *nrp1a* (described in [64]). Matched heads from embryos of the same genotype were pooled and were processed for immunohistochemistry.

### Whole-mount immunohistochemistry

Immunohistochemistry was performed as previously described [48]. Larvae were fixed in 4% paraformaldehyde in PBS and dehydrated in methanol. To visualize Citrine positive axons, larvae were permeabilized in acetone for 20 min at −20°C and stained with goat anti-GFP (1:100; Rockland Immunochemicals, 600-101-215) and donkey anti-goat IgG Alexa Fluor 488 (1:500; Invitrogen). Propidium iodide staining was performed following secondary antibody treatment as described by Brend and Holley, 2009 [75] with the exception that larvae were not treated with RNase. Confocal microscopy was performed on an inverted Leica SP5 using a 40× oil-immersion lens. Stacks were acquired through the entire OB with optical sections taken 1 μm apart.

### Whole-mount fluorescent in situ hybridization

Double-label in situ hybridization was performed using antisense digoxigenin (DIG) RNA probes to guidance genes and fluorescein-labeled probes to OR subfamily genes as previously described [36, 64], with the exception that RNase treatment was not performed after probe removal. Following probe hybridization and removal, embryos were incubated in anti-DIG-POD (1:500; Roche, 11207733910) and the DIG label was amplified using the cyanine 5-coupled tyramide kit to label axon guidance genes. OR transcripts were detected using anti-fluorescein-POD (1:500; Roche, 11426346910) and the fluorescein label was amplified using a fluorescein-coupled tyramide kit (PerkinElmer, NEL741001KT). Propidium iodide labeling and imaging were performed following the second tyramide amplification [75]. The plasmids used to make probes targeting *nrp1a* and *nrp1b* were as described by Dell et al., 2013 [76] and Taku et al., 2016 [64]. For *robo2* (refseq accession number NM_131633.1, nucleotides 3445–4382) and *pcdh11* (refseq accession number XM_005173190.3 nucleotides 220–839) sequences were amplified from 48hpf zebrafish cDNA and cloned into pcRII-TOPO Dual promoter TA cloning kit (Invitrogen, K460001) for probe synthesis. Full-length probes were used in all hybridization experiments except for *robo2* where the probe was hydrolyzed prior to incubation.

### Quantification of targeting errors

The number of OBs containing axons terminating in either individual protoglomeruli or non-protoglomerular regions were counted. Axons were scored as projecting to a particular protoglomerulus only if they terminated in that protoglomerulus and not if they passed though it *en route* to another location. The percentage of OBs with axons in each protoglomerulus was computed and a two-tailed Fisher’s exact test was used to determine statistical significance. Error bars represent standard error of the mean.
